# The impact of fatigue severity and depression level on the quality of life in individuals with Parkinson’s disease in Taiwan

**DOI:** 10.3389/fpsyt.2024.1309989

**Published:** 2024-07-18

**Authors:** Hung-Yu Lin, Yi-Tung Lin, Chou-Ping Chiou, Hsueh-Hsing Pan

**Affiliations:** ^1^ School of Medicine, College of Medicine, I-Shou University, Kaohsiung, Taiwan; ^2^ Division of Urology, Department of Surgery, E-Da Cancer Hospital, I-Shou University, Kaohsiung, Taiwan; ^3^ Department of Nursing, E-Da Hospital, I-Shou University, Kaohsiung, Taiwan; ^4^ School of Nursing, I-Shou University, Kaohsiung, Taiwan; ^5^ School of Nursing, National Defense Medical Center, Taipei, Taiwan; ^6^ Department of Nursing, Tri-Service General Hospital, Taipei, Taiwan

**Keywords:** fatigue severity, depression level, quality of life, Parkinson’s disease, social support

## Abstract

**Background:**

Patients diagnosed with Parkinson’s disease undergo alterations in physical, psychological, and social functionality, with the psychological domain being particularly predisposed to inducing fatigue and depressive symptoms. Understanding the alterations occurring within a patient’s body and mind and how these influence their overall quality of life is crucial.

**Purpose:**

This study sought to explore the predictive capacity of fatigue severity, the presence of depressive symptoms, and diverse demographic factors on the quality of life among individuals with Parkinson’s disease.

**Methods:**

A cross-sectional correlational study was conducted at a teaching hospital in southern Taiwan. The research utilized a questionnaire survey to interview 133 study participants, focusing on the Quality of Life Scale, Depression Scale, Fatigue Severity Scale, Social Support Scale, and demographic attributes.

**Results:**

A total of 130 valid questionnaires were obtained. The results showed that Hoehn and Yahr stage, fatigue severity, and depression status could predict quality of life, explaining 51.1% of the total variance. These findings suggest that patients at advanced Hoehn and Yahr stages, experiencing more severe fatigue, and exhibiting higher levels of depression, tended to report a lower overall quality of life. Our findings suggest that, in addition to Hoehn and Yahr stage, the severity of fatigue and levels of depression significantly impact the quality of life in individuals with Parkinson’s disease.

**Conclusion:**

Nurses need to understand the “stressful life events” and the changes in appearance and physical function that patients with Parkinson’s disease face due to chronic degenerative diseases. Hence, apart from addressing patients’ physiological needs, healthcare professionals should also offer appropriate care for psychological issues, such as depressive symptoms. Encouraging patients to participate in “counseling groups” can further bolster their social support networks, enhancing their overall well-being and addressing comorbidities associated with chronic degenerative diseases.

## Introduction

### The prevalence of PD

Parkinson’s disease (PD) represents a significant global health challenge, particularly impacting the elderly demographic. Over recent decades, advancements in medical technology have contributed to an extended average lifespan among older individuals, resulting in a society characterized by a predominant presence of aging adults. According to health insurance statistics data, as of March 27, 2022, there were 77,428 PD patients in Taiwan. In terms of the total population, approximately three out of every ten thousand individuals have PD. In terms of age groups, the highest prevalence was observed among those aged 71 to 80, followed by those aged 81 to 90 and 61 to 70. PD is a prevalent neurodegenerative condition in Taiwan, impacting around 1–2% of the population aged 65 and above (1). According to statistics from the Ministry of the Interior, as of March 2023, the proportion of people aged 65 and above in Taiwan has reached 17.74%, surpassing the United Nations’ definition of a “senior nation,” which is seven percent. Projections indicate that by 2025, one in every five individuals in Taiwan will be elderly, surpassing the 20% mark and ushering in a “superaged society” (2).

### The importance and significance of QoL in PD patients

In the rapidly evolving healthcare environment, quality of life (QoL) has become an essential indicator of healthcare. PD is a common neurodegenerative disease that causes chronic debilitation. Although advanced medical interventions can alleviate the symptoms of PD, it cannot be cured. The disease hampers patients’ mobility, daily activities, and overall physical and social functioning, significantly affecting their QoL (3). Hence, enhancing the patients’ QoL for patients has gained recognition as a crucial component of PD treatment. It is anticipated that the results of this study can help provide valuable insights for healthcare professionals, better understand patients with PD, and serve as an indicator and reference for assessing clinical care outcomes, enabling effective implementation of appropriate measures to improve the condition of PD patients and enhance their QoL.

### Correlation between fatigue severity, depression level, and QoL

The prevalence of fatigue in PD stands at 50% (4). Research indicates that the progressive degeneration induced by PD is irreversible, subjecting patients to enduring physical, mental, and social discomfort and stress throughout the protracted treatment process. Consequently, the commonly and persistently experienced symptom of “fatigue” has been identified. Furthermore, studies have consistently revealed that fatigue is one of the most common nonmotor symptoms of PD, with the potential to diminish QoL (5). The prevalence of depression among PD patients is reported to be 38%. Depression profoundly affects patients’ QoL and can potentially lead to disability. Early treatment for depression may improve the QoL of PD patients (6). The progressive and irreversible nature of PD forces patients to confront future lifestyle changes, often resulting in feelings of depression, denial, and helplessness. These psychological barriers can accelerate the progression of the disease (7).

### Demographic characteristics of PD patients as the relevant factors

PD ranks as the second most prevalent neurodegenerative disease among elderly individuals, next to Alzheimer’s disease. Age stands out as the most critical risk factor, while the primary pathological changes predominantly occur in the basal ganglia, specifically the degeneration of the substantia nigra and corpus striatum. This results in a decrease in dopamine secretion or an imbalance in the ratio of acetylcholine to dopamine, affecting motor disturbances (8). Patients with PD often grapple with life challenges arising from symptoms such as tremors, muscle stiffness, and gradual loss of coordination, typically manifesting initially on one side of the body. As the condition progresses, the other side of the limbs may also exhibit symptoms, leading to an unstable trunk and difficulty walking, speaking, and swallowing (9). PD is categorized into five stages based on the Hoehn and Yahr scale, commonly abbreviated as the H & Y stage: Stage 1: Mild symptoms occur on only one side of the body, with slight tremors in the limbs that do not affect daily life; Stage 2: Symptoms occur on both sides of the body, affecting posture and gait; Stage 3: Balance problems when walking or standing, noticeable slow movements, and partial limitations in daily life; Stage 4: Stiffness and coordination problems occur, requiring walking aids and everyday life assistance from others; Stage 5: Unable to walk and reliance on lying in bed or sitting on a wheelchair, everyday life depends on the care of others (1). Their QoL tends to decline as the H & Y stage advances in PD patients. PD patients eventually experience abnormal movements such as muscle stiffness, involuntary tremors, and slowed movements (10). The motor dysfunction caused by PD, such as bradykinesia, rigidity, freezing of gait, resting tremors, depression, and fatigue, negatively impact patients’ QoL (11).

In examining the demographic characteristics of PD patients, we have found that age, gender, and employment status significantly affect their QoL (12). Studies also highlight that PD’s progressive and worsening symptoms lead to declining self-care abilities and increased motor impairments, disease severity, and comorbidities. Therefore, motor impairments, disease severity, and the number of comorbidities all affect the QoL of patients (4).

Given the need for further research in Taiwan regarding the QoL and related factors in PD patients, this study aims to achieve three key objectives: 1. Assess the current QoL status of PD patients; 2. Explore the relationships between demographic characteristics, social support, fatigue, depression, and QoL in PD patients, and 3. Identify predictive factors that influence the QoL of PD patients.

## Materials and methods

### Design and participants

This cross-sectional correlational design was conducted at a teaching hospital in the southern region of Taiwan. The study population comprised PD patients who met specific inclusion criteria and voluntarily agreed to participate. The inclusion criteria encompassed the following: (1) a diagnosis of primary PD, (2) no prior surgical treatment for PD, (3) mental alertness without psychiatric disorders or dementia, (4) the ability to communicate with the researchers in Mandarin Chinese or other mutually understandable languages, and (5) provision of informed consent after the research purpose was explained. The exclusion criteria are (1) patients who have previously undergone surgical treatment for Parkinson’s disease. (2) adults who are unable to provide informed consent. (3) individuals with unstable medical conditions.

The sample size estimation for this study was based on power analysis using Cohen’s method (13). For regression analysis, with the power set at 0.80, effect size at 0.15, α level at 0.05, and 12 predictors, the estimated sample size was 127 individuals. An additional 5% was estimated to account for invalid questionnaires, resulting in 133 participants.

### Instruments

Data collection instruments were a demographic questionnaire, the Interpersonal Support Evaluation List, the Fatigue Severity Scale, the Geriatric Depression Scale, and the PD Questionnaire. The items on the demographic questionnaire included age, gender, religious beliefs, education level, marital status, socioeconomic status, number of comorbidities (including cardiovascular diseases, genitourinary system diseases, gastrointestinal diseases, respiratory diseases, tumors, and others), and the H &Y stage. The attending physician assessed the H &Y stage of PD.

### Interpersonal support evaluation list

This scale primarily assesses the perceived availability of assistance from spouses, family members, friends, healthcare professionals, colleagues, and others for PD patients. It encompasses four dimensions: emotional support, appraisal support, informational support, and tangible support (14). Scoring is conducted on a four-point Likert scale (0–3), with higher scores indicating better social support. The Chinese version of this 16-item social support scale, translated by domestic scholars, demonstrated a two-week test-retest reliability of 0.77 (n=64) and Cronbach’s α of 0.81 (N=129) (15). In this study, the questionnaire achieved a Cronbach’s α of 0.79.

### Fatigue severity scale

This scale comprises nine items and evaluates the severity of fatigue symptoms on a scale ranging from 1 to 7, where 1 indicates “strongly disagree” and 7 indicates “strongly agree.” The final score is the average, with a score of ≥4 indicating significant fatigue. Regarding reliability, Cronbach’s alpha was found to be 0.88. In terms of validity, the Fatigue Severity Scale (FSS) demonstrated a statistically significant positive correlation with the Visual Analogue Scale (VAS) (r = .68, p < 0.001) (16). Due to its good reliability and validity, this tool has been utilized by researchers abroad in assessing fatigue severity in Parkinson’s disease patients and its impact on daily life (17). The Chinese version of the scale translated by (18) demonstrated good internal consistency with a Cronbach’s α of 0.92.

The FSS has been found to have a significant positive correlation with the Visual Analog Scale (VAS) measuring fatigue severity (r = .68, *p* < 0.001). The Cronbach’s α of the questionnaire in this study was 0.95.

### Geriatric Depression Scale

The Geriatric Depression Scale Short-Form Version (GDS-SF) consists of 15 items. It is widely utilized for depression screening in diverse settings, including community, acute medical, or long-term care facilities for older adults. It is designed to be free from bias related to race or region. The scale uses a binary response format (“yes” or “no”) to self-report “feelings over the past week.” A score of 0 is assigned to “no” responses, and a score of 1 is assigned to “yes” answers. The total score on this scale ranges from 0 to 15, with higher scores indicating a greater likelihood of experiencing depressive symptoms. A score of 5–8 indicates mild depression, 9–11 indicates moderate depression, and 12 or higher indicates severe depression. The sensitivity of the cutoff point of this scale is 96.3%, and the specificity is 87.5%. Additionally, its Cronbach’s α was reported as.84 (19), while the questionnaire achieved a Cronbach’s α of 0.73 in this study.

### Parkinson’s disease questionnaire

The PDQ-39 is a disease-specific questionnaire developed to assess the QoL of individuals with PD (20). It consists of eight dimensions: mobility, activities of daily living, emotional well-being, stigma, social support, cognitive impairment, communication, and bodily discomfort. Participants rate the items on a Likert scale ranging from 0 to 4, indicating the increasing frequency of occurrence (21). Designed by Peto et al., this questionnaire has demonstrated strong reliability (ICCs = 0.72) (22). In its Chinese version, the PD QoL questionnaire has reliability (Cronbach’s α = 0.54- 0.90) and validity (Spearman’s rank correlation coefficients = 0.25- 0.83) (23). The questionnaire contains 39 items across eight domains related to daily life activities, mobility, cognition, social support, communication, emotions, shame, and physical discomfort. Total scores are weighted and transformed to fall within a range of 0–100, where lower scores indicate a higher QoL.

### Procedure

The researcher interviewed PD patients who met the inclusion criteria and willingly agreed to participate, as provided by the neurosurgery outpatient clinic. Before administering the questionnaire, the researchers explained the research purpose, process, and time required to the participants and obtained informed consent from the participants or their family members. The researcher verbally presented each questionnaire question, allowing the participants to provide their answers. Disease-related information was collected from the medical records. The interview time was about 30–40 minutes.

### Ethical considerations

The hospital’s Institutional Review Board approved the study. The researchers explained the study purpose and procedure to the participants and obtained their written informed consent by signing a consent form before data collection. Participants were informed that their involvement in the study was voluntary and that they had full authority to withdraw at any time without giving a reason. They were also assured that their information would be treated with the utmost confidentiality and presented in a way that preserved their anonymity entirely. To ensure anonymity, all personal identifiers were eliminated from the survey, and the obtained data were securely stored in a computer protected by a password. Ethical considerations were upheld throughout the research process.

### Data analyses

Data entry and statistical analyses were performed using IBM SPSS Statistics version 26. Descriptive statistics were analyzed, including frequency distribution, percentages, means, and standard deviations. Inferential statistics involves the analysis of multiple regression.

## Results

### Demographic characteristics of respondents

The researchers reviewed each questionnaire in this study, and 130 valid responses were obtained. The predominant age group among the study participants was over 50 years old, with an equal gender distribution. The education level was predominantly elementary school graduation, and the economic status was moderate. Most participants were married, and a significant proportion were unemployed or retired. The H&Y stage predominantly fell within the second and third stages, while the number of comorbidities was typically either zero or one, as illustrated in **Table 1**.

**Table 1:** Frequency distribution and percentages of demographic characteristics of PD patients (N=130).

Variable	*n*	%
Gender		
Male	65	50.0
Female	65	50.0
Age		
<50	6	4.6
50–64	39	30.0
65–74	45	34.6
>75	40	30.8
Education		
Incomplete elementary school	33	25.4
Elementary school	36	27.7
Junior high school	11	8.4
High school	24	18.5
College or above	26	20.0
Marital status		
Unmarried	18	13.8
Married	112	86.2
Religious belief		
Taoism	43	33.1
Buddhism	65	50.0
Christianity	3	2.3
Other religions	4	3.1
None	15	11.5
Economic status		
Insufficient	19	14.6
Sufficient	104	80.0
Adequate	7	5.4
Employment status		
Employed	19	14.6
Unemployed	66	50.8
Retired	45	34.6
Number of comorbidities		
0	46	35.4
1	49	37.7
2	28	21.5
3	5	3.9
4	2	1.5
H & Y stage		
Stage 1	7	5.4
Stage 2	48	36.9
Stage 3	43	33.1
Stage 4	26	20.0
Stage 5	6	4.6

### The distribution of social support, fatigue severity, depression level, and QoL

**Table 2** presents the mean scores for social support (M = 28.95, SD = 6.40), fatigue severity (M = 4.05, SD = 1.88), with all participants scoring ≥4, quality of life (M = 54.60, SD = 36.13), and depression level, (M = 7.52, SD = 2.66), with ten participants classified as having severe depressive symptoms, accounting for 7.7% of the sample.

**Table 2:** Social support, fatigue, depressive symptoms. and QoL (N = 130).

Item	Mean	*SD*	N	*%*
**Social support**	28.98	6.40		
**Fatigue severity**	4.05	1.88		
>4			57	43.8
=4			17	13.1
**Quality of life**	54.60	36.13		
**Depressive level**	7.52	2.66		
mild (5–8)			66	50.8
moderate (9–11)			38	29.2
Severe (≥12)			10	7.7

### Predictive factors of QoL in PD patients

Multiple regression analysis was employed to examine the predictive factors of QoL in PD patients. The regression model underwent assessment for multicollinearity, with the maximum variance inflation factor (VIF) for each variable found to be less than 5, affirming the appropriateness of the regression model. As depicted in **Table 3**, the findings of this study confirm the H&Y stage, severity of fatigue, and level of depressive symptoms as significant influencers of QoL in PD patients. The model accounted for a substantial 51.1% of the variance in QoL, underscoring that patients with lower H&Y stages, less severe fatigue, and lower depressive symptoms had better overall QoL. In summary, this study establishes that H&Y stage, fatigue severity, and level of depressive symptoms are significant predictive factors of QoL.

**Table 3:** Results of multiple regression for QoL of PD patients (N=130).

Variables	B	SE	β	t	p	95% CI	
						Lower	Upper
**H & Y stage**	15.116	2.656	.428	5.691	<0.001	9.844	20.388
**Depressive level**	3.681	1.048	.279	3.513	<0.01	1.601	5.762
**Fatigue severity**	0.624	0.159	0.292	3.937	<0.001	0.309	0.939

## Discussion

### Demographic characteristics of PD patients

This study demonstrates an equal distribution of male and female patients, aligning with the 2021 Taiwan National Health Insurance database statistics (male: 47%, female: 53%) (24). We also confirmed that PD mainly affects individuals over 50 years old, in line with prior research indicating that the incidence and prevalence of PD increase with age (25).

### Distribution of social support, fatigue severity, depression level, and QoL

The study results revealed a mean social support score of 28.95 (SD = 6.40), indicating moderate to high levels of social support, consistent with findings from the study (26). The mean fatigue score was 4.05 (SD = 1.88), with all participants scoring ≥4, signifying significant fatigue. This finding aligns with similar levels of fatigue reported in studies of Parkinson’s disease patients (27). The mean score for quality of life was 54.60 (SD = 36.13), comparable to findings from research on PD patients (28). The mean depression score was 7.52 (SD = 2.66), consistent with the average GDS scores reported in the study of PD patients (29).

### Predictive factors of QoL in PD patients

This study affirms that fatigue severity in PD patients significantly affects their QoL. This finding is consistent with an investigation that suggests that PD patients experience physical, psychological, and emotional changes that reduce their functional abilities and affect their QoL (30). PD patients experience fatigue due to the physical and cognitive effort required in daily activities, resulting in reduced daily activities, coping abilities, physical function, self-roles, and social function. This reliance on others significantly impacts the QoL of patients and caregivers (31). Another study also noted that fatigue in PD patients substantially hinders daily functioning, affecting emotional well-being and overall QoL (32).

The findings of this study emphasize that depression has a notable impact on the QoL of individuals with PD, consistent with previous research highlighting the link between depression severity and reduced QoL (33). These results also align with another prior study (14), which revealed that depression in PD patients not only impairs physiological, psychological, and social functioning but also exerts adverse effects on overall QoL. Furthermore, depression may complicate disease management, exacerbate other physical symptoms, impair cognitive function, and cause distress for both patients and their caregivers.

This study also establishes a noteworthy correlation between PD patients’ H&Y stage and QoL. As the severity and stage of H&Y increase, QoL decreases, consistent with previous studies (34). PD patients grapple with motor abnormalities such as muscle stiffness, tremors, and slowed movements, leading to significant inconvenience and impacting their daily QoL. Furthermore, disease severity, physical disability, and decreased independence in daily living emerged as pivotal factors affecting PD patients’ QoL (35, 36).

### Limitation and recommendation

To the best of our knowledge, this is one of the few studies that specifically investigate the impact of PD on QoL in the Taiwanese population. We have included several understudied variables in PD research, such as fatigue and depression on QoL. These factors are often overlooked in PD research but may have significant implications for patient care and treatment. Previous studies have shown that fatigue impacts QoL in PD (37), while depression also affects QoL in PD (38). Our study goes beyond assessing merely the physical symptoms of PD, incorporating a thorough evaluation of psychological facets. This holistic approach provides a more complete picture of the disease’s impact on QoL.

Our findings may also have implications for treatment approaches. The high impact of non-motor symptoms on QoL in our study suggests that treatments targeting fatigue and depression may improve QoL for PD patients. However, this study still has its limitations as follows, and we have also proposed suggestions for future research.

1. This study was conducted in a teaching hospital in southern Taiwan. The results from a single-center study may not be generalizable to other settings due to differences in patient populations, healthcare providers, and institutional practices. Additionally, the study design was cross-sectional. As for the lack of longitudinal follow-up, we cannot assess changes over time or determine the durability of the observed effects.

We hope to address these limitations in future research by collaborating with other centers and implementing a longitudinal study design. Alternative scales, like the Unified Parkinson’s Disease Rating Scale (UPDRS), exist to track the longitudinal progression of Parkinson’s Disease (PD). UPDRS is the commonly used scale employed in clinical PD studies due to its widespread utilization. Further research should prioritize long-term monitoring of PD patients to detect symptoms early, enabling prompt intervention to slow disease progression and preserve patients’ QoL.

Acknowledging these limitations is crucial for interpreting study results accurately and informing future research directions. We believe that acknowledging these limitations does not diminish the value of our findings but rather provides a more balanced and accurate interpretation of our results.

2. The relationships between fatigue, depression, and QoL are complex and multifaceted in various health conditions, with each factor potentially influencing and being influenced by the others. Understanding the potential mechanisms underlying the relationship between fatigue, depression, and QoL is crucial for providing a more comprehensive understanding. Psychological factors, including coping strategies and perceptions of illness, also shape the relationship between these variables.

This study primarily investigates whether fatigue and depression will affect QoL. Subsequent research could delve deeper into investigating whether there are mediating or moderating effects between these variables.

Understanding these mechanisms is crucial for developing targeted interventions that address both fatigue and depression to improve QoL in individuals with chronic health conditions. Further research is needed to elucidate these relationships and develop effective interventions fully.

3. PD is a neurodegenerative disorder that significantly impacts the QoL of patients. Fatigue and depression are common non-motor symptoms in PD patients that can severely affect their daily life. Therefore, it is crucial to develop effective interventions to manage these symptoms. specific targeted interventions, including exercise programs, cognitive behavioral therapy (CBT), support groups, professional lectures, and group recreational activities, as potential strategies for managing fatigue and depression in PD patients. The ultimate goal is to improve PD patients’ QoL. Future research should focus on evaluating the effectiveness of these interventions and identifying additional strategies for improving the QoL of PD patients. It is also essential to explore how these interventions can be best tailored to individual patient’s needs and preferences. This could involve investigating different types of exercise programs, different approaches to CBT, and different formats for support groups and recreational activities. Ultimately, further research aims to provide evidence-based recommendations for managing fatigue and depression and improving QoL in PD patients. Focusing on patient-centered care and evidence-based interventions can significantly improve the QoL of PD patients. This approach underscores the importance of a holistic, patient-centered approach in managing PD, emphasizing evidence-based practice.

## Conclusion

The main findings of this study reveal that fatigue and depression significantly impact the QoL of PD patients. Therefore, addressing how to improve their fatigue and depression to enhance patients’ QoL is an urgent issue for healthcare professionals. Implement a multidisciplinary approach involving healthcare professionals from various specialties, such as neurology, psychiatry, physiotherapy, and occupational therapy. Collaboration among these professionals can lead to comprehensive care plans tailored to the individual needs of PD patients. Provide education and resources to PD patients and their caregivers about the importance of managing fatigue and depression. Empowering patients with knowledge about coping strategies, lifestyle modifications, and available support services can improve their ability to manage these symptoms and enhance their quality of life. Offer psychological support and counseling services to PD patients experiencing fatigue and depression. Cognitive-behavioral therapy, mindfulness-based interventions, and support groups can provide valuable coping skills and emotional support.

To further enhance the quality of care, it is beneficial to incorporate the concepts of Parkinson’s disease-related depression and fatigue into the curriculum of “Parkinson’s disease and care.” As for future research directions, it may be worthwhile to explore interventions such as “peer support groups” or “home care” aimed at reducing the severity of fatigue and depression in PD patients, ultimately leading to an improvement in their overall QoL.

Disease knowledge and communication skills should be emphasized in medical and nursing education. Education and clinical practice are interconnected, and through continuing nursing education, healthcare professionals can better support PD patients in coping with the multifaceted challenges posed by physical, psychological, social, and environmental aspects caused by the disease. Based on the findings of this study, healthcare professionals in clinical practice can gain a deeper understanding of the fatigue experienced by PD patients at various stages of H&Y. Multidisciplinary collaboration and rehabilitation programs should be integrated to increase patients’ sensitivity to movement responses. Regular professional lectures or group recreational activities should be organized to reduce depressive symptoms in PD patients while enhancing their daily functional abilities.

Support ongoing research efforts to understand better the underlying mechanisms of fatigue and depression in Parkinson’s disease and to develop novel interventions and treatment strategies. Continued innovation in this field is essential for improving PD patients’ outcomes and QoL.

## Data availability statement

The raw data supporting the conclusions of this article will be made available by the authors, without undue reservation.

## Ethics statement

The studies involving humans were approved by E-Da Hospital’s Institutional Review Board. The studies were conducted in accordance with the local legislation and institutional requirements. The participants provided their written informed consent to participate in this study.

## Author contributions

HL: Writing – original draft, Resources. YL: Writing – original draft, Conceptualization. CC: Writing – review & editing, Supervision, Software, Resources, Project administration, Methodology, Formal analysis, Conceptualization. HP: Writing – review & editing, Validation, Supervision, Resources, Conceptualization.
